# Implementation of an interactive organ donation education program for Dutch lower-educated students: a process evaluation

**DOI:** 10.1186/s12889-020-08900-5

**Published:** 2020-05-20

**Authors:** Esther Steenaart, Rik Crutzen, Nanne K. de Vries

**Affiliations:** Department of Health Promotion, CAPHRI Maastricht University, P.O. Box 616, 6200 MD Maastricht, The Netherlands

**Keywords:** Education program, Organ donation registration, Adolescents, Implementation, The Netherlands, Vocational education

## Abstract

**Background:**

As organ donation registration rates remain low, especially among lower-educated people, it is important to support this group in making their registration decision. To prepare lower-educated students in the Netherlands for making a well-informed decision, an interactive educational program was developed. We aim to understand both the (quality of) implementation as well as to contextualize the effects of this program in a lower-educated school setting.

**Methods:**

The process evaluation was part of a Cluster Randomized Controlled Trial, in which 11 schools for Intermediate Vocational Education throughout the Netherlands participated. Teachers who taught a course on Citizenship delivered three intervention elements (i.e. video fragments and discussion, quizzes with tailored feedback and an exercise filling out a registration form) to their students. Implementation was assessed by interviews with teachers, questionnaires from students, logbooks from teachers and user data from Google Analytics.

**Results:**

The program was well received and implemented, but on-the-spot adaptations were made by teachers to fit their students better. Within the lower-educated target group, differences between students are high in terms of active participation, reading abilities, knowledge and attention span. The program fit well within their regular teaching activities, but the topic of organ donation is not always prioritized by teachers.

**Conclusions:**

We see opportunities to disseminate the program on a larger scale and reach a group that has been neglected in organ donation education before. Within the program, there are possibilities to increase the effectiveness of the program, such as alternative delivery methods for the elements with a lot of text, the addition of booster sessions and guidelines for teachers to adapt the program to students of different levels within Intermediate Vocational Education. Moreover, in order to have an impact on a national level, strategies need to be employed to reach high numbers of students and, therefore, support on a higher level is needed (both within schools and at policy level).

**Trial registration:**

Dutch Trial Register, NTR6771. Prospectively registered on 24 October 2017.

## Background

Shortage in the availability of organs for transplantation is an important public health challenge. Even though organ donation rates vary dramatically from country to country (due to e.g. registration systems or transplant coordination), there is a universal shortage and the global need for organs continues to grow [1, 2]. In the Netherlands, the number of people that are waiting for an organ is also growing, causing patients to die or suffer other consequences while waiting [3]. While these problems are no different than in many other countries, the Dutch situation is quite unique. The donor legislation is different from other countries, and so are policy and education initiatives.

Currently, an opt-in registration system is used in the Netherlands. Next to registration as a donor, people also have the opportunity to register another decision, such as registration as a non-donor or leaving the decision to next of kin. However, as an opt-in system is based on voluntary registration, people also have the option not to choose, and a large proportion of the population still makes use of this option [4]. Many efforts have been taken to encourage people to make a decision regarding their donor record and actually register their decision. Increasing the number of registrations is important to help more patients and can also take away uncertainties around the death of someone without a donor record. Siminoff and Lawrence showed that having knowledge of a patient’s preference to donate, increases the likelihood of donating and the family’s satisfaction with the decision [5].

At the age of 18, Dutch citizens are asked to make a decision about their donor status by the Ministry of Health, Welfare and Sports. As only about 1 in 3 adolescents respond every year, there is clearly a need for support in this decision-making process [6]. Teaching adolescents about organ donation has the potential to fulfil this role and increase registration rates. Several studies have shown higher levels of knowledge, a more positive attitude towards organ donation, a higher willingness to donate and/or higher intentions to register a decision in participants of such programs [7–9]. However, these programs have mostly been directed at schools for higher educational levels. The school system for lower levels of education is different (in terms of students, teachers, and organizational structure) and existing organ donation education programs have not reached these specific schools so far (and might also not be suitable for this setting). This is worrying, as lower-educated people are found to have a less positive attitude about organ donation [10–12], spend less time looking for information about organ donation on the Internet, talk less about organ donation with others, are less aware of news coverage on organ donation [10] and are less often registered [13]. Therefore, we designed an interactive educational program, especially for a lower-educated target group and school setting, focused on supporting adolescents in making a well-informed decision about organ donation and encouraging them to register this decision.

This program was implemented in a school setting and delivered by teachers. Teachers, therefore, play a large role in the quality of implementation. Next to evaluating program effects, a process evaluation has become an increasingly important part of a comprehensive program evaluation [14] to understand both the (quality of) implementation as well as to contextualize the possible effects of the program. The current study focuses on describing and evaluating the implementation of this educational program for lower-educated adolescents, using multiple methods.

## Methods

A process evaluation was conducted of an educational program about organ donation at 11 schools that implemented the program. This process evaluation is part of a Cluster Randomized Controlled Trial (CRCT) evaluating the effectiveness of the organ donation program. More details about the development of the program, the study design and methods [15] and the effect evaluation [16] can be found elsewhere. The study was approved by the Ethics Committee of the Faculty of Health, Medicine and Life Sciences on 23 October 2017 (reference number: Steenaart/231017) and prospectively registered at the Dutch Trial Register (NTR6771; https://www.trialregister.nl/trial/6557). This article was written in accordance with the COREQ checklist (see Additional file 1).

### Program

**Fig. 1:**
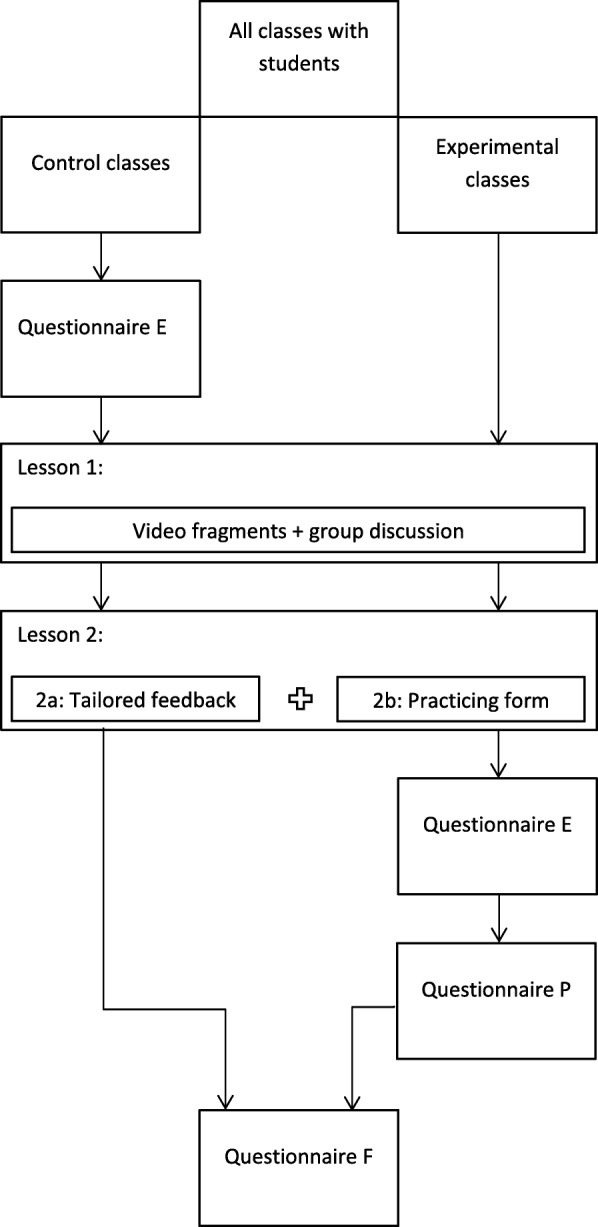
Design of the evaluation study, *Questionnaire P* questionnaire process evaluation. *Questionnaire E* questionnaire effect evaluation. *Questionnaire F* questionnaire follow-up measurement

The original program was designed by Reubsaet and colleagues to support high school students in making a decision about organ donation (regardless of this being as a donor or not) and to encourage them to register this decision [8, 17, 18]. The program was developed based on the social cognitive theory of Bandura [19]. Several formative studies focusing on determinants and pilot tests led to the final educational program, which was then successfully implemented in a high school setting. Later, the program was translated from a paper-based to a web-based version. This version was adjusted to make it suitable for a lower-educated target group, based on a determinant study among this group [20]. This lower-educated target group concerns students that left high school after four years and continue their education in Intermediate Vocational Education (IVE). In principle, these students are above the age of 16, so written informed consent was only obtained from the students themselves, not from their parents, as approved by the Ethical Committee. Within IVE, students can choose from a variety of disciplines. There are four levels (of which level 1 is the lowest and level 4 is the highest) and the educational level of this group in general is lower as opposed to college or university education. The effectiveness of this adjusted program was evaluated in a CRCT with post-test only (Fig. 1).

**Fig. 2:**
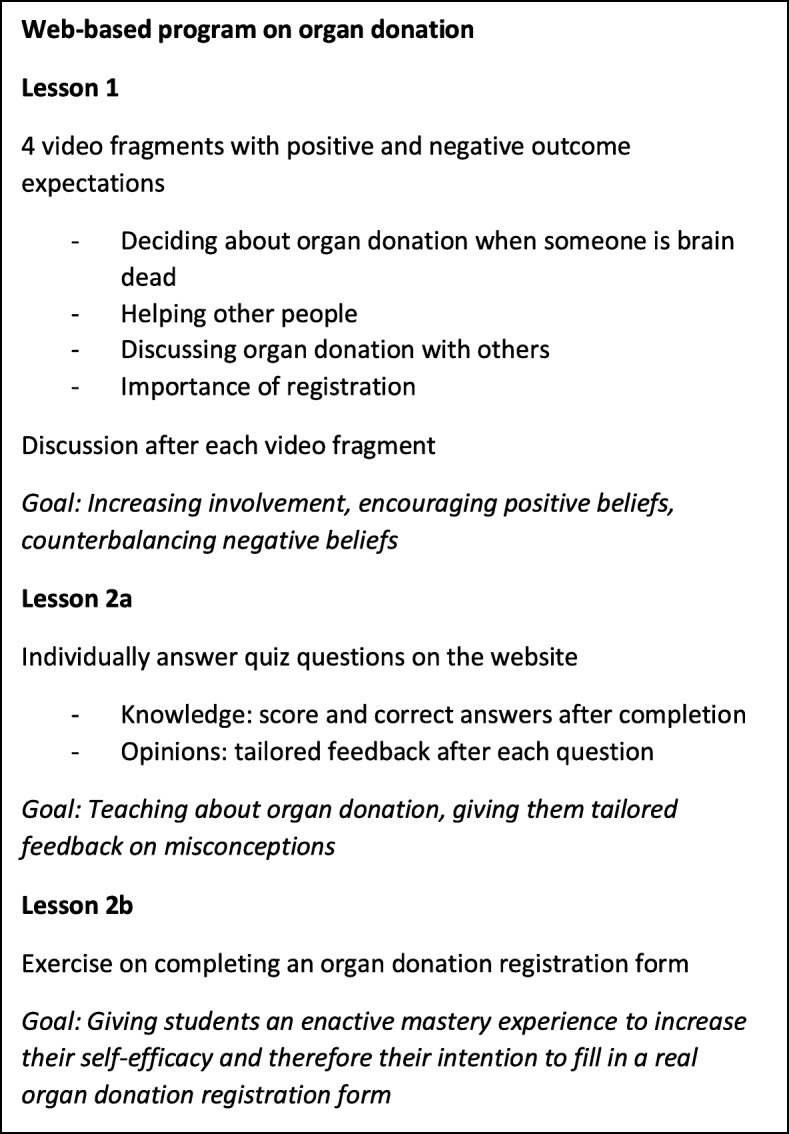
Content of the educational program about organ donation

The program consisted of a website with three elements to be completed in a school setting during two 50-min-lessons. A combination of individual and plenary activities was used. Figure 2 provides an overview of the lessons and goals of the program.

### Recruitment and participants in trial

Teachers from schools for Intermediate Vocational Education were invited to participate in the study by e-mail. These were typically teachers providing a course on Citizenship. Teachers that agreed to participate were asked to draw up an inventory of their classes in terms of the number of students, educational level and study discipline. The classes were randomized into control and experimental, after which the teachers received all materials (questionnaires for the students, logbooks and teacher manual) and a personalized link for every class. The program was offered to the experimental group and after measurement also to the control group. At the individual level, no inclusion or exclusion criteria were set, only on class and school level, so everyone participated in the program, regardless of age. After receiving the data, there appeared to be 5 students of 15 years old in our data (out of 1170 in total). They were excluded from the data analysis. At class level, a variety of study disciplines and levels of education were included. Classes from level 1 (entry level) were excluded from participation because of the large variety in cognitive abilities at this level. Later, teachers from all schools who completed the program and returned the questionnaires and logbooks were invited over e-mail for a telephone interview to elaborate on their experiences in the classroom. No additional exclusion criteria were set.

### Data collection

Data collection took place between October 2017 and October 2018. The implementation of the program was evaluated both during and after the delivery of the program. During the delivery, user data were collected by means of Google Analytics. After delivery, students in the experimental groups filled in a questionnaire about the program they received. All other data was collected in both experimental and control groups. Teachers were asked to fill in a logbook after each lesson. Teachers who returned the logbooks were invited to participate in a telephone interview. Reasons not to participate were related to a lack of time.

The interviews were conducted by researcher ES. ES was a female PhD researcher, trained in doing qualitative research. Teachers were familiar with her as she also provided the support during recruitment and implementation. In the beginning of the interview ES informed the teachers about the goals of the interview. The interviews lasted approximately 20 min and were the main data source for this study. Interviews usually took place within a few weeks after implementation, but near the end of the trial (and also the end of the school year), recruiting for participation in an interview became more difficult. One interview was planned after the summer vacation, but this teacher clearly had difficulties remembering details of the implementation, which was a natural moment to stop recruiting.

### Measurements

Implementation of the educational components was measured using six constructs, inspired by the model of Steckler and Linnan [14]. Table 1 provides an overview of abovementioned constructs, the definitions used and the instruments used to measure these. The interviews were the main data source in this study, complemented by user data from Google Analytics, logbooks from teachers and student questionnaires.

**Table 1:** Constructs of implementation with their definitions and instruments

Construct	Definition	Measurement instrument
*Reach*	The extent to which the target group is reached: - Number of schools reached - Number of participants reached	Google Analytics
*Dose delivered*	The proportion of program components actually delivered by teachers: - Number of components delivered	Logbooks teachers Interviews teachers
*Dose received*	The extent to which students engage with the program: - Active participation during the lessons - Appreciation of the program (components)	Logbooks teachers Interviews teachers Questionnaire Google Analytics
*Fidelity*	The extent to which the program was implemented as intended: - Compliance to the manual - Completeness of deliverance of the components	Logbooks teachers Interviews teachers Google Analytics
*Context*	Environmental aspects that could affect the implementation: - Compatibility with the existing curriculum - Teachers’ capacity to implement the program - Support by the schools’ board or colleagues	Interviews teachers
*Reasons for fidelity and dose*	Reasons for (not) implementing the program (components) as intended: - Facilitators to implementation - Barriers to implementation	Logbooks teachers Interviews teachers Questionnaire

#### Interviews

To learn more about teachers’ experiences and barriers and facilitators to the implementation, semi-structured interviews were used. Data from the logbooks were used during the interview with that particular teacher, to give them the opportunity to elaborate on answers they gave. The interviews were based on a topic list, focusing on teachers’ experiences, dissemination and innovation characteristics (the latter derived from the Diffusion of Innovations Theory by Rogers [21]). The full topic list can be found in the Open Science Framework repository (https://osf.io/sk6mr/).

#### Google analytics

Participants’ behaviour on the website (at an aggregate level) was assessed by Google Analytics. Teachers were provided with a personalized link per class, which enabled us to trace user behaviour back to specific classes (i.e., not to individuals within that class). The user data gave us insight in when the website was visited by which classes, how participants behaved on the website (e.g. how long they stayed on a certain page), whether all elements were completed and gave us an indication whether elements were used in an individual or plenary session. User data cannot explain why teachers and students behaved in a certain way. This was mainly explored during the interviews.

#### Logbooks

Teachers were asked to fill out a logbook after each lesson to learn more about what components they delivered, how much time they spent on the different components, how satisfied they were with the components and any other comments they wanted to share. Whether components were delivered, was assessed by a simple yes/no checklist. Each component was scored on a scale from 1 to 10.

#### Questionnaires

To include students’ experiences in the evaluation as well, students in the experimental groups filled in a questionnaire after completing the program. They first indicated whether they were present during lesson 1 and 2. Their appreciation for the different components was then assessed on a 7-point Likert scale (totally disagree to totally agree), by asking them whether they felt a particular component was fun and whether they felt that component was informative. They also scored the program as a whole on a scale from 1 to 10 (in line with the Dutch grading system). Finally, they had the opportunity to mention things they specifically liked about the program and give recommendations for improving the program.

### Analyses

The interviews were audio-taped, notes were made during the interview and a transcript was written from each interview afterwards. Nvivo was used to code and group the qualitative data from the interviews and the open-ended questions in the logbooks and questionnaires. Thematic analysis was used to analyse the data [22]. After getting familiar with the data, a code tree was made based on which the first interviews were coded. After this first round, themes in the code tree were reviewed by two researchers and adaptations were made. All interviews were then coded based on this new code tree. The final code tree can be found on OSF: https://osf.io/sk6mr/. The coding of the interviews was individually done by two independent researchers, after which the differences were identified and consensus was reached. The quantitative data (logbooks, questionnaires and Google Analytics) were analysed using SPSS24, by means of frequencies and descriptives.

## Results

### Reach

A total of 11 schools with 1170 students participated in the study. Of those students, 601 (i.e., those in the experimental group) filled out the process evaluation questionnaire. Of these, 67.1% was female and 40.6% were enrolled in a study in the area of health care and well-being. They were on average 18.09 years old. Thirty teachers kept track of a logbook during and after the implementation of the program, leading to a total of 72 logbooks being returned. Ten teachers were interviewed after completing the implementation of the program. The interviewed teachers were from eight different schools from different geographical regions. Three were male, seven were female, and their age ranged from 23 to 62. Their teaching experience varied from 1 year to 30 years. As not all teachers made use of the right link (i.e. to allow tracing back to class-level), results on reach were only derived from returned forms and interviews and not based on Google Analytics.

### Dose delivered

Logbooks from teachers showed that the video fragments and group discussions were delivered by 98.6% of them (Table 2). Deliverance of the second lesson was a little lower, with 87.7% of the teachers letting their students work with the quizzes on the website. Finally, 55.1% let the students practice with a paper-based registration form in lesson 2b, while 39.6% used the digital form, and 88.5% provided the students with information about the registration form. In total, 96.2% of the teachers delivered at least one element of lesson 2b. Teachers were divided on whether paper-based or digital materials work better. One teacher commented that everything an IVE-student does, is online nowadays. On the other hand, teachers mentioned that students benefit from physical materials as well:

*“And in the end I printed the registration form, because that often helps, that they then have something tangible”- Teacher, 38, female.*

**Table 2:** Deliverance of each lesson according to teachers’ logbooks

Lesson	Component delivered (% yes)
**Lesson 1**	
- Showing video fragments	98.6%
- Facilitating group discussion	98.6%
**Lesson 2a**	
- Let students make quizzes	87.7%
**Lesson 2b**	
- Practicing on paper	55.1%
- Practicing online	39.6%
- Provide information about form	88.5%

### Dose received

#### Active participation

Teachers were divided about the active participation of their students. In some classes, students were very enthusiastic, had vivid discussions and asked a lot of questions:

*“They were all very interested and then of course there is a group that says ‘We are not allowed to donate because of our religion’. But then they really discuss it respectfully and that leads to very nice conversations ( …*) *So you have really nice questions and discussions.” – Teacher, 45, female.*

*“The atmosphere in the class was very quiet and attentive” – Student, 16, female, level 2.*

In other classes, it was harder to motivate students, as they were not convinced of the relevance of (education about) the topic or simply not interested. Some teachers suggest that this depends on the study discipline. Students enrolled in a health care program were more interested in the topic than some other classes:

*“Well, what should be noted; these are technical guys, so they are – they just want to know how something works, that’s it. They are not going to philosophize for a long time about feelings and things like that. I can imagine that this is different in other groups” – Teacher, 52, female.*

Some teachers said personal relevance was very important to motivate their students as well. They suggested that this could be embedded more in the program, but also personal stories from teachers or students led to more active participation:

*“And I have to say, I have a friend, for example, who has a new kidney, so I also told them some of my own stories and they listened carefully to that. So that then makes it easier for me, and well, the involvement of students as well I think, when you have your own stories” – Teacher, 38, female.*

#### Appreciation of the program (components)

In the interviews, teachers were in general enthusiastic about the program. They saw the relevance of teaching about organ donation, especially for the age group (around 18 years old) they work with. Moreover, the program was easy to use for them as teachers, but also easy to understand for the students. Further, they liked the variety of components and the fact that the program was ready-to-use. Students rated the program as a whole with a 7 out of 10, which is considered a very adequate mark in the Netherlands. They also appreciated the variety of components:

*“I liked that there was a lot of variety and that they asked about your opinion a lot and that it makes you think about it” – Student, 16, female, level 4.*

##### Lesson 1

Teachers were most positive about lesson 1, scoring it a 7.5 out of 10. Also students were most enthusiastic about this lesson (Table 3). Teachers said the video fragments were appealing and they liked that they involved different perspectives on organ donation (patient, surviving relative, etc). Using videos works well with their students and the actors and stories were easy to relate to:

**Table 3:** Students’ evaluations of the different components and the program as a whole

Lesson	Score (1–7 scale)
**Lesson 1**	
Video fragments	
- Fun	4.4 ± 1.6
- Informative	4.6 ± 1.6
Group discussion	
- Fun	4.8 ± 1.6
- Informative	4.5 ± 1.6
**Lesson 2**	
Quizzes	
- Fun	4.2 ± 1.6
- Informative	4.1 ± 1.6
Registration exercise	
- Fun	3.9 ± 1.8
- Informative	3.9 ± 1.8
**Entire program (1–10)**	7.0 ± 1.2

*“The videos worked well. I remember them leaving an impression. Especially one, I remember, that they, I believe it was an interview at a cemetery, or a grave, that someone said “Well, this is a nice way to start a lesson”. But then they were like: “But why? Why is this on the screen?” So I immediately had their attention. And it made use of three people with different perspectives. (…*) *That was just very good and worked fine” – Teacher, 38, female.*

Students were in general positive about the videos and liked the discussions followed by these.

*“I liked that different people were interviewed, to make us hear different stories” – Student, 17, male, level 4.*

However, they also commented on the quality of the videos, the presenter and editing of the videos:

*“I found repeating the text with white words on the screen a little childish” – Student, 21, male, level 4.*

##### Lesson 2a

The second lesson (2a) was rated with a 6.3 by teachers and received critical comments during the interviews. Students were moderately positive about this component (Table 3). The main problem that teachers described was that students did not read the questions or answers properly and therefore finished within a few minutes. Moreover, some teachers found it difficult that they had no idea what their students were doing, which made it hard for them to follow up on it or answer questions. Finally, they believed the entire second lesson was too theoretical and could benefit from some more interaction.

*“Only the quiz didn’t reach its full potential, I wrote that down a couple of times. ( …*) *I have no idea what they do with the questions. So you can’t really follow up on that. And for them it sometimes was unclear what the results actually meant. And they passed through it quickly. It took them less than 10 minutes per quiz.” – Teacher, 23, female.*

##### Lesson 2b

Lesson 2b was rated with a 6.9 by the teachers, and least appreciated by the students (Table 3). Most teachers did see the added value of paying attention to the registration form, but found it hard to motivate the students. They mentioned that students sometimes felt that they already knew how it worked or did not understand why they had to actually fill it out, as it was not for real. Further, as it was the last component, at the end of a lesson, students were not able to maintain their focus. A few teachers shared examples of things that went wrong when filling out the form, emphasizing the importance of the lesson:

*“There was even a boy who said he filled out the form on paper at home and that his father did a final check and that he then appeared to have made a mistake. So that was very nice. ( …*) *Because the question ‘which organs do you want to donate or not’, well, he mixed those up” – Teacher, 52, female.*

*“We have that registration form – for level 2 students, that’s a really good exercise, as they don’t even know their social security number or even their zip code so to speak, really, sometimes they are so ignorant.” – Teacher, 26, female.*

### Fidelity

#### Compliance to manual

All teachers that were interviewed expressed being happy with the manual. It gave them clear guidance, some knowledge about the topic and some said it made them more confident. Most said they took the time beforehand to read it thoroughly. Moreover, the frequently asked questions that were added in the manual were appreciated.

*“I found the guidelines, the manual that was added, very nice. Also with all information, a few of the most frequently asked questions. A few times I thought, ‘oh I have some additional questions’, but then I didn’t e-mail the contact persons that were in there, but I could have. But no, that was very nice. Also the extra bits of information. Yes, that gave sufficient guidance.”- Teacher, 29, female.*

Even though all teachers made use of the manual, not all components were implemented as intended. Teachers made on-the-spot adaptations to make it fit their group or schedule better. Some teachers fit all information in 1 lesson and some spread it out over 3 lessons. Especially differences in the level of education of the students seemed to be an important reason to make adaptations. For the higher educated classes within IVE, teachers sometimes added extra components themselves (e.g. a more elaborate discussion based on statements they found online or asking students to write a summary of what they learned), while for the lower educated classes, they made the lessons shorter (e.g. by watching less videos or only explaining the registration form instead of making them practice with it).

*“Yes, it’s too many, 4 [videos] is really too many. The attention span of IVE-students is not that big, so at some point, when you also want to do those discussions afterwards, ( …*) *so I intentionally shortened these because otherwise I would’ve lost them at the third video already. So the first 3 are great, the one with the doctor as well, but that one is just, it also takes like 5 minutes or so, that one is too long. At least, for my classes, they are too long” – Teacher, 29, female.*

*“The lessons could have been shorter, because at some point I didn’t have any concentration left, while the topic was very interesting”- Student, 19, female, level 3.*

#### Completeness of deliverance of components

Also from the information from the logbooks and Google Analytics it became apparent that not all components were delivered in a complete manner (Table 4). According to the logbooks, teachers spent 50 min on the first lesson, which was exactly as intended. However, Google Analytics showed that the average time spent on the page with the video fragments was slightly under 20 min (Table 5). This might mean that teachers spent more time on introducing the topic and the group discussions and thus chose not to show all videos. This also was confirmed by some teachers in the interviews:

*“I did watch a few of the videos, but not all of them. So I gave a few as an example, and then of course especially the ones where youngsters were involved, because that fits their experience world.”- Teacher 37, male.*

**Table 4:** Time spent on each component according to teachers’ logbooks

Lessons	Time spent
Lesson 1	50 min
Lesson 2a	24 min
Lesson 2b	17 min

**Table 5:** Time spent on each page and number of page views according to Google Analytics

Page	Number of page views	Average time per page
Video fragments	438	19:24
Introduction page quizzes	1238	00:12
Explanation page quizzes	1172	00:14
Quizz 1: What do you know about organ donation?	1973	2:19
Quizz 2: What is your opinion about organ donation?	1967	1:05
Practicing with registration form	476	9:14

Teachers said lesson 2a took 24 min on average, but the Google Analytics data showed that the time actually spent on the quizzes was much lower. The first quiz took students 2:19 min to complete, while the second quiz only took them 1:05 min to finish. This suggests that students did not read the questions properly, did not think about their answers and/or did not read the tailored feedback. Teachers mentioned this in the interviews as well:

*“Then I think they didn’t really read what the outcome was, only the score they got and that’s it. Students don’t read any more these days, it’s really useless” – Teacher, 28, female.*

Teachers indicated they spent on average 17 min on lesson 2b. As this lesson is not necessarily done on the web page, it is difficult to confirm this. The way in which lesson 2b was delivered differs between teachers, as some used the paper-based version, others the digital version and others only showed it to them, without making them do the exercise.

The number of page views gives an indication of whether components were delivered in a plenary or individual session (Table 5). It seems like most teachers delivered the video fragments and registration form in a plenary session, while the quizzes were done individually.

### Context

#### Compatibility with existing curriculum

Teachers agree that the program fits very well within the course Citizenship and then especially within the Vitality dimension, but also other options were suggested, such as linking it to their age (what changes in your life when you turn 18?) or the political dimension (as in 2018 a law change on organ donation was accepted). Many teachers mentioned that Citizenship does not have a lot of requirements, which can both be a positive and negative thing. It means that there is quite some room for topics of choice, but on the other hand, there are so many topics to choose from, that organ donation is not always a priority:

*“At the moment we have – here I go again with my schedule – but we limited ourselves with the dimension of Vitality, because of time constraints. So we discuss alcohol, and that is – well, I teach technical boys who on top of that want to become car mechanics, so that is an important topic. (...) And another topic we chose is STDs and, well, I always try to find some time for a healthy diet, but then often my time is up”- Teacher, 52, female.*

Some teachers found it very easy to find time for two lessons, while others mentioned that they have very limited time for Citizenship in general and therefore two hours was too much. This probably highly depends on the school and curriculum. Most teachers said that if they know about the program in advance, it is not too difficult to find time for it.

At the moment, there is no program or information about organ donation in the regular Citizenship methods, but some teachers already spent time on it in previous years, as they find it an important topic. In those cases they usually shared a personal story with the students or invited a guest speaker.

*“It’s not a topic that is usually part of Citizenship so to say, it’s not part of the requirements within the domain, but yes, I always discuss it. So I think it’s definitely useful to do something with that” – Teacher, 45, female.*

#### Teachers’ capacity to implement the program

Teachers generally see themselves as being capable to implement the program. They said the program was easy to use and the instructions were clear. However, as organ donation is not their expertise, they also encountered some challenges. Teachers sometimes got questions from students they did not know the answer to. They handled these questions very differently:

*“It [eligibility for donation] depends on age and some other factors. And then they were quite interested, like, where can I find that? I said, well I think you should just Google it. I mean, you can just find that on the Internet” – Teacher, 62, male.*

*“And I just said in the beginning, like “Guys, I did go through a couple of frequently asked questions beforehand, but I also don’t know everything, so maybe I’ll look something up” – Teacher, 52, female.*

Teachers were also aware that organ donation is a sensitive topic to discuss. Most teachers did not necessarily see this as a problem, but all dealt with it in their own way. Some teachers found it difficult to get a feeling of how far they could push and talk about taboos. Others said that the sensitivity of the topic led to very open discussions:

*“Well, I know that one girl knows she needs a donor lung to prevent her from dying young. But we’ve talked about that in the past as well. So, it is tough, but doable. I am at least - but that also depends on the teacher I think, how he/she deals with that - I am just very relaxed and open about it and I’m also very young. So I’m very close to them. So that makes a difference. So you will get those questions or comments any way, but if you open up and are vulnerable, students will do the same” – Teacher, 28, female.*

#### Support by the schools’ board or colleagues

As mentioned before, Citizenship is a course in which teachers have a lot of freedom to teach about topics they find important. Implementing it on a school level might therefore be challenging. However, some teachers commented that they think it would be of added value to implement the program on a school level.

*“Yes, well actually, I don’t know how much power you have ( …*) *But I think that if you want it to be very powerful, it is utopian what I’m going to say now, but that it will become an obligatory component of the curriculum. ( …*) *So the government needs to invest in that, in education, like hey, there’s one period in their entire school career, that when they’re 18, we are going to spend time on that.” – Teacher, 37, male.*

No other teachers mentioned involvement of the school board, but sometimes discussed it among colleagues or in Citizenship meetings.

### Reasons for fidelity and dose

#### Facilitators to implementation

If both students and teachers saw the relevance of learning/teaching about organ donation, this facilitated the implementation. There were several ways in which the program became more relevant. For instance, one teacher chose to deliver the program around Christmas and another teacher suggested that it might be good to link it to the yearly national Donor week. Further, around the time of implementation, an upcoming new donor law got a lot of media attention:

*“For me, it was a godsend at that time. Because it was very topical then. So I thought like, how am I going to address that in my Citizenship classes? Because, coincidentally, we were also just dealing with Vital Citizenship, so yes, that fits fine of course. ( …*) *So in that sense: timing was perfect” – Teacher, 38, female.*

Another factor that helped in making it relevant for the students is their age. During the year after they turn 18, they receive a letter in which they are asked to make a choice regarding organ donation.

*“Because it’s of course also the age that fits this very well. So there are 16, 17, 18 year-olds in the first year, that’s where I implemented it, and yes, for 16-year-olds it might not concern them yet, but then there’s also 18-year-olds there, so you can ask “Who is already 18 here? Do you remember getting that letter?” And to a 16 or 17-year-old you could say: “Well, now you know what to expect when you turn 18”. So that’s a nice preparation, fits their age as well. So also without topicality, you could make it relevant to address” – Teacher, 38, female.*

Other facilitating factors for implementation were that the program was easy to use for both students and teachers and that effects were observable. They mentioned that they had interesting discussions about it, students clearly had more knowledge after, that they sometimes said they talked about home or even registered:

*“Yes, some of them changed their opinions. In the beginning they often said “oh I don’t want that”, and now they started thinking “Oh well, maybe I do”. Like: “I don’t know yet”, but it’s not a harsh no at least. So that’s really nice to see, that they start thinking critically. (…*) *They at least became aware of the fact that they have a choice. And that they have to think about it themselves, if you want to influence the outcome. That’s one thing. And secondly, that they started thinking critically. So they might not have an answer yet, but are dealing with it” – Teacher, 38, female.*

*“I learned a lot and now I know more about donor registration and what happens after that with your organs and body” – Student, 17, female, level 4.*

#### Barriers to implementation

One of the most important barriers to implementation, as mentioned before, was the fact that IVE-students do not read materials that well. Some teachers said that they just forget and immediately want to do things instead of reading about it. Others are just discouraged when seeing a lot of text. Sometimes this is because of more serious problems such as low-literacy:

*“In level 2, and especially in level 1 but we don’t even teach them, there are many people with low-literacy” – Teacher, 52, female.*

Teachers therefore recommend to stick to the content of the quizzes, but then delivering it in a more interactive way. This could also help overcoming another barrier, which is the low attention span of students in IVE (see: Fidelity: compliance to manual).

Two practical issues were mentioned by teachers, that made the implementation more difficult. One teacher mentioned she had a large group, which made it hard to involve everyone, while another teacher mentioned that she struggled with absenteeism of students during the first lesson. These are issues that cannot be prevented, but suggestions on how to deal with this could be added to the manual.

Finally, different cultural or religious backgrounds were mentioned as being either a facilitator or a barrier. One teacher said that having more students with a migration background made things a little bit more challenging, while others said that the mixed backgrounds of their students facilitated lively discussions:

*“I have to say that I found it very interesting because our school is very mixed, so we have around, well I don’t know, maybe 40 to 50 different cultures here. So that leads to very nice discussions” – Teacher, 45, female.*

### Future use

All teachers were positive towards future use, sometimes even actively asking questions about this in the end of the interviews themselves. They seem willing to make time available for this topic in the future. Some of them want to follow up in the same classes as well. There was also a specific request by some teachers to make a follow-up lesson to be able to repeat some things and elaborate in the same classes some time later.

*“Look, this time I had a gap to fill, but I think that I’ll make time for this in the future” – Teacher, 29, female.*

They believed that especially colleagues teaching Citizenship would be interested in using the program as well. However, they also mentioned that you probably highly depend on the teacher that you encounter. Some teachers prefer to spend time on other topics, or might just not be enthusiastic about organ donation themselves.

*“I think colleagues in Citizenship would [be interested in using the program], yes. However, it does depend on the teacher. Because I’m a little bit more involved in those things, that I find these things important and others have their focus on other topics. That’s a thing with this subject [Citizenship]. That it’s very dependent on the teacher” – Teacher, 23, female.*

One teacher (with a background in health care) was afraid that the program might be difficult to teach for people without a background in health care, but other teachers did not conform that. One other teacher recommended to send reminder e-mails, as teachers tend to run from pillar to post.

### Recommendations

Both teachers and students gave recommendations for further improvement of the program. Teachers mainly emphasized the need of different versions or options for the different educational levels within IVE. They argued that level 2 classes cannot be compared to level 4 classes in terms of active participation, attention span, reading and knowledge.

*“I personally solemnly believe in different programs, with the same subjects and the same goals for both groups. Because for level 2, these kind of topics don’t concern them yet, unless they have personal experiences with it” – Teacher, 52, female.*

In practice, this could mean that students in level 2 classes need some more (inter) active methods and something that makes it personally relevant for them. For level 4 classes this could mean that there are optional assignments or more in depth information for those who are interested. Teachers commented that the program was not challenging enough for the higher achieving classes or students:

*“Maybe it would be nice – because I talked about that with colleagues recently, we are working with differentiation in our classes, that especially those lessons with the quizzes and information that many students were like “I already knew that”, that you might be able to add a bonus component or so. (…*) *Many students said at the beginning of the program “Yes, I have an opinion and I can support that because it is what it is”. So it would be a challenge to pose some questions about that. That would be nice” – Teacher, 29, female.*

This also became clear from the comments that students added to their evaluation form. For some students the program was elaborate enough (or even too long). On the other hand, many students commented that they would have liked even more information:

*“I’d like more information about the process of organ donation, how it actually works” – Student, 21, female, level 4.*

Students also commented that the program was very serious and sometimes even boring.

*“Maybe it can be delivered in a more playful way. It was very formal and serious” – Student, 24, female, level 3.*

Teachers gave some suggestions to make the program more attractive for students. One teacher suggested involving a celebrity in the videos, who spoke about organ donation on a television show earlier.

Finally, some students found the program too much pro-donation. They liked having different perspectives on the topic, but suggested that the perspective of someone who is against it or decided not to become a donor could be added.

*“The videos and registration form make you feel bad if you don’t want to be an organ donor” – Student, 16, female, level 4.*

*“The only comment some students had was that they felt it was a little bit forced on them. That’s what one or two students said. But I said “Any way you slice it, they ask you to think about it and about the choice you’ll make” – Teacher, 37, male.*

## Discussion

An interactive organ donation education program was developed to support lower-educated adolescents in making a well-informed decision about organ donation. The program proved to be successful in increasing (determinants of) registration intentions. The present study focused on the process of implementation of this program in an IVE school setting. While the teachers’ perspective was the most important data course, also the students’ perspective and user data from the website were taken into account. By combining different measurement tools, a complete overview of the implementation process was given and findings could be confirmed, leading to a higher validity of conclusions [14, 23, 24].

The program as a whole was delivered quite well by the teachers. Lesson 1 was implemented with high fidelity and appreciated by both teachers and students. Lesson 2 (both the quizzes and registration forms) was appreciated less and also not used as intended. Teachers and students commented that it was too theoretical and that more interaction would be appreciated. It was noted that the second lesson required a lot of reading, which appeared to be a problem for the lower-educated target group. This could partly be due to high rates of low literacy within IVE: 1 out of 3 students in level 2 and 1 out of 7 in level 3 (there is no data available regarding level 4) [25].

During the implementation of a similar program in a high school setting, Reubsaet and colleagues also saw that lesson 2b was implemented least often [8]. Most important reasons for this were a lack of time and the fact that teachers did not seem to see the usefulness and importance of the training. These results were taken into account when adapting the program to a lower-educated target group by shortening the quizzes to ensure there was time left for the registration exercise and by adding a section about the importance of this lesson in the manual. It seems that these changes were successful in overcoming the barriers as teachers did not mention these issues during the interviews anymore.

However, during the implementation in a lower-educated target group, new barriers emerged. While teachers were convinced of the importance, students were not. Moreover, not all students were capable to fill out the form themselves and their attention span was low at the end of the lesson. This probably explains why some teachers only gave an explanation about the form, but did not do the exercise. So, even though giving students an enactive mastery experience is traditionally seen as most influential to increase self-efficacy [26], this might not play out in this context and target group. While the content of the lesson is still very relevant, the way of delivering this should be reconsidered.

Overall, relevance of the topic and the program (elements) for both teachers and students appeared to be an important factor for implementing the program (well). When conducting the study, the relevance of teaching about organ donation was high as a law change about organ donation (to be implemented in 2020) was widely discussed in the news. However, teachers commented that in the future, it might be difficult to prioritize teaching about organ donation over other important (health-related) topics, such as safe sex or drug use. This can be challenging as organ donation registration serves the society as a whole, but does not directly impact the well-being of their students, especially not in the short run. Therefore, for future dissemination of the program, support on a higher level should be explored.

To increase the attractiveness and relevance of the program for students, different options are open. One option could be a collaboration with a celebrity, with the main goal of attracting students’ attention. When combined with accurate and balanced information, celebrity advocacy has the potential to influence health behavior [27, 28], and is seen as a powerful tool for both health literacy and health promotion [29]. As young adults have a more positive attitude regarding celebrity engagement than older people [30], this could be a promising addition to a program in an adolescent target group. It is important to then choose a celebrity that students can easily identify with, as this is an important mediator of celebrity effects on students’ attitudes and behaviors [31, 32]. Attitudes, values and behaviors are likely influenced by celebrity issues, when someone feels personal identification with that celebrity [33]. As the target group is very diverse, it will be a challenge to choose a person that most students feel connected to.

This diverse target group gave us an important insight: even when tailoring a program to a specific target group, there might be differences within this group that cannot be ignored. Teachers made on the spot adaptations, which is inevitable when implementing school-based interventions [34, 35]. In this real life setting, an intervention needs to fit the context and the students (in this case especially different educational levels and study disciplines) [36, 37]. Moreover, teachers might make adaptations to fit their own pedagogical approach or to incorporate their own stories and experiences [38]. This brings us to the ongoing fidelity-adaptation debate. While traditionally fidelity is seen as critical for success, adaptations also seem beneficial. It increases local ownership and sustainability [39], may improve the engagement of students and teachers who adapt are possibly more motivation [40], creative and effective in implementing [41]. Adaptations should therefore not be discouraged, but guidelines on how and where to make those adaptations in order to still ensure effectiveness of the program are needed [42, 43].

Another desirable adaptation could be the addition of follow-up interventions, or so called ‘booster’ interventions. In the effectiveness study of the same program, it was already noted that decision-making regarding organ donation takes time and is very complex [7]. Especially in this situation, where for most students it was their first encounter with the topic of organ donation, it takes time to form an opinion and act on it. In this process evaluation, several teachers asked for extra materials as they also felt the need to revisit the topic later or to come to more in-depth understanding and discussion. Booster interventions give the opportunity to reinforce and build on information to suit students’ age and development [44]. Booster sessions are common in the field of health promotion, including education about drugs [45], life style programs [46] or worksite interventions [47]. However, as organ donation registration is a one-time behaviour, booster sessions are not relevant anymore for students who already registered their decision. This was also mentioned by some students who were already registered during the initial implementation of the program. The application of booster sessions in the context of organ donation education should therefore be further investigated, to ensure relevance for all students.

### Limitations

Overall, when reading the results, one should be aware that there was a risk of sample bias in this study. Teachers that are enthusiastic about the topic of organ donation and who are open to new educational programs might be more likely to use the program and to agree to the interview afterwards. This was also stressed by teachers themselves during the interviews, by explaining that implementation of such programs highly depends on the individual teacher. It is important to keep in mind that teachers who are against organ donation or the upcoming law change might not be willing to teach about it or influence the implementation in a negative way.

Another possible limitation concerns the variability in time between the implementation and the interviews with teachers. Often, the interviews were conducted within a short amount of time after the implementation, but sometimes this was not possible due to busy schedules. Recall bias could have occurred, which possibly led to the loss of details. While this limitation should be acknowledged, this is not likely to have influenced the results in general, as the overall experience will not have changed over a few weeks. It might however have influenced the richness of interviews in the cases with a longer time interval.

## Conclusions

This interactive organ donation education program was well received and implemented within a lower-educated school-setting. Therefore, we see opportunities to disseminate the program on a larger scale and reach a group that before has been neglected in organ donation education. Within the program, there are possibilities to increase the effectiveness of the program, such as alternative delivery methods for the elements with a lot of text, the addition of booster sessions and guidelines for teachers to adapt the program to students of different levels within IVE. Moreover, in order to have an impact on a national level, strategies need to be employed to reach high numbers of students and therefore support on a higher level is needed (both within schools and at policy level).

## Supplementary information

**Additional file 1.** COREQ Checklist.

## Data Availability

The datasets and materials generated and analyzed during the current study are available in the Open Science Framework repository, https://osf.io/sk6mr/.

## Abbreviations

IVE: Intermediate Vocational Education
CRCT: Cluster Randomized Controlled Trial

## References

[1] Rudge C, Matesanz R, Delmonico FL, Chapman J (2012). International practices of organ donation. Brit J Anaesth.

[2] Rithalia A, McDaid C, Suekarran S, Myers L, Sowden A (2009). Impact of presumed consent for organ donation on donation rates: a systematic review. BMJ..

[3] Nederlandse Transplantatiestichting (2017). Jaarverslag 2017: Verbinden voor leven. [Annual report 2017: Connecting for life].

[4] Centraal Bureau voor de Statistiek. 6,3 miljoen personen in donorregister. [6.3 million people in the donor registry]. https://www.cbs.nl/nl-nl/nieuws/2018/32/6-3-miljoen-personen-in-donorregister. Published 9 August 2018. Accessed 6 Feb 2019. [in Dutch].

[5] Siminoff LA, Lawrence RH (2002). Knowing patients’ preferences about organ donation: does it make a difference?. J Trauma Acute Care.

[6] Ministerie van Volksgezondheid, Welzijn en Sport. Respons aanschrijvingen - jongeren. [Response notifications – youngsters] https://web.archive.org/web/20170314103917/https://www.donorregister.nl/cijfers/responsaanschrijvingen/jongeren Accessed 4 March 2019. [in Dutch].

[7] Cárdenas V, Thornton JD, Wong KA, Spigner C, Allen MD (2010). Effects of classroom education on knowledge and attitudes regarding organ donation in ethnically diverse urban high schools. Clin Transpl.

[8] Reubsaet A, Brug J, Nijkamp MD, Candel M, Van Hooff J, Van den Borne H (2005). The impact of an organ donation registration information program for high school students in the Netherlands. Soc Sci Med.

[9] Vinokur AD, Merion RM, Couper MP, Jones EG, Dong Y (2006). Educational web-based intervention for high school students to increase knowledge and promote positive attitudes toward organ donation. Health Educ Behav.

[10] Cox D (2005). Naar een goed gevoel: communicatie en niet-registratie bij donorvoorlichting. [towards a good feeling: communication and non-registration in donor education].

[11] Nijkamp Marjan, Hollestelle Marianne, Zeegers Maurice, van den Borne Bart, Reubsaet Astrid (2008). To be(come) or not to be(come) an organ donor, that's the question: a meta-analysis of determinant and intervention studies. Health Psychology Review.

[12] Wakefield C, Watts K, Homewood J, Meiser B, Siminoff L (2010). Attitudes toward organ donation and donor behaviour: a review of the international literature. Prog Transplant.

[13] Peters F, Schmeets H (2015). Bevolkingstrends. Het donorregister: wie doet mee en wie niet? [Population trends. The donor registry: who is in and who is not?].

[14] Steckler AB, Linnan L, Israel B (2002). Process evaluation for public health interventions and research.

[15] Steenaart E, Crutzen R, Candel MJJM, de Vries NK (2018). A web-based education program to encourage organ donation registration among lower-educated adolescents in the Netherlands: study protocol for a cluster randomized controlled trial. Trials..

[16] Steenaart E, Crutzen R, Candel MJJM, De Vries NK (2019). The effectiveness of an interactive organ donation education intervention for Dutch lower-educated students: a cluster randomized controlled trial. Trials..

[17] Reubsaet A, Brug J, De Vet E, Van Den Borne B (2003). The effects of practicing registration of organ donation preference on self-efficacy and registration intention: an enactive mastery experience. Psychol Health.

[18] Reubsaet A, Brug J, Kitslaar J, Van Hooff J, Van Den Borne H (2004). The impact and evaluation of two school-based interventions on intention to register an organ donation preference. Health Educ Res.

[19] Bandura A (1986). Social foundations of thought and action: a social cognitive theory.

[20] Steenaart E, Crutzen R, de Vries NK (2018). Complexity of organ donation registration: determinants of registration behavior among lower-educated adolescents. Transplant Proc.

[21] Rogers EM (2010). Diffusion of innovations.

[22] Guest G, MacQueen KM, Namey EE (2011). Applied thematic analysis.

[23] Adami M, Kiger A (2004). The use of triangulation for completeness purposes. Nurse Res.

[24] Hussein Ashatu (2009). The use of Triangulation in Social Sciences Research. Journal of Comparative Social Work.

[25] Christoffels I, Groot A, Clement C, Lam J-F. Preventie door interventie. Literatuurstudie naar lees- en schrijfachterstanden bij kinderen en jongeren: prevalentie, relevante factoren en mogelijke interventies. [Prevention through intervention. Literature study about reading and writing deficiency of children and youngsters: prevalence, relevant factors and possible interventions]. 's-Hertogenbosch: Expertisecentrum Beroepsonderwijs; 2017. [in Dutch].

[26] Bandura A (1996). Self-efficacy: the exercise of control.

[27] Larson RJ, Woloshin S, Schwartz LM, Welch HG (2005). Celebrity endorsements of cancer screening. J Natl Cancer I.

[28] Sabel MS, Dal CS (2016). Trends in media reports of celebrities’ breast cancer treatment decisions. Ann Surg Oncol.

[29] Hoffman SJ, Tan C (2015). Biological, psychological and social processes that explain celebrities’ influence on patients’ health-related behaviors. Arch Public Health.

[30] Panis K, Van Den Bulck H (2012). Celebrities’ quest for a better world: understanding Flemish public perceptions of celebrities’ societal engagement. Javnost-Public..

[31] Brown WJ, Basil MD, Bocarnea MC (2003). The influence of famous athletes on health beliefs and practices: mark McGwire, child abuse prevention, and androstenedione. J Health Commun.

[32] Brown WJ, De Matviuk MAC (2010). Sports celebrities and public health: Diego Maradona's influence on drug use prevention. J Health Commun.

[33] Yoo W (2016). The influence of celebrity exemplars on college students' smoking. J Am Coll Heal.

[34] Durlak JA, DuPre EP (2008). Implementation matters: a review of research on the influence of implementation on program outcomes and the factors affecting implementation. Am J Commun Psychol.

[35] Ringwalt CL, Ennett S, Johnson R (2003). Factors associated with fidelity to substance use prevention curriculum guides in the nation's middle schools. Health Educ Behav.

[36] Chambers DA, Glasgow RE, Stange KC (2013). The dynamic sustainability framework: addressing the paradox of sustainment amid ongoing change. Implement Sci.

[37] Hansen WB, Pankratz MM, Dusenbury L (2013). Styles of adaptation: the impact of frequency and valence of adaptation on preventing substance use. Health Educ Res.

[38] Lendrum A, Humphrey N, Greenberg M, Shute RH, Slee PT (2016). Implementing for success in school-based mental health promotion: the role of quality in resolving the tension between fidelity and adaptation. Mental health and wellbeing through schools: the way forward.

[39] US Department of Health and Human Services (2002). Finding the balance: program fidelity and adaptation in substance abuse prevention.

[40] Botvin GJ (2004). Advancing prevention science and practice: challenges, critical issues, and future directions. Prev Sci.

[41] Dusenbury L, Brannigan R, Falco M, Hansen WB (2003). A review of research on fidelity of implementation: implications for drug abuse prevention in school settings. Health Educ Res.

[42] Hall GE, Hord SM (2019). Implementing change: patterns, principles, and potholes.

[43] Center for Substance Abuse Prevention (2002). Finding the balance: program fidelity and adaptation in substance abuse prevention.

[44] White D, Pitts M (1997). Health promotion with young people for the prevention of substance misuse.

[45] McBride N (2003). A systematic review of school drug education. Health Educ Res.

[46] Alharbi M, Gallagher R, Kirkness A, Sibbritt D, Tofler G (2016). Long-term outcomes from healthy eating and exercise lifestyle program for overweight people with heart disease and diabetes. Eur J Cardiovasc Nur.

[47] Goldberg L, Lockwood C, Garg B, Kuehl KS (2015). Healthy team healthy U: a prospective validation of an evidence-based worksite health promotion and wellness platform. Front Public Health.

